# Hydroxytyrosol and Cytoprotection: A Projection for Clinical Interventions

**DOI:** 10.3390/ijms18050930

**Published:** 2017-04-28

**Authors:** Francisca Echeverría, Macarena Ortiz, Rodrigo Valenzuela, Luis A. Videla

**Affiliations:** 1 Nutrition Department, Faculty of Medicine, University of Chile, Independencia 1027, Independencia, Santiago 8380453, Chile; francisca.echeverria@med.uchile.cl; 2 Nutrition and Dietetics School, Faculty of Health Sciences, Catholic University of Maule, Merced 333, Curicó 3340000, Chile; mortiz@ucm.cl; 3 Molecular and Clinical Pharmacology Program, Institute of Biomedical Sciences, Faculty of Medicine, University of Chile, Independencia 1027, Independencia, Santiago 8380453, Chile; lvidela@med.uchile.cl

**Keywords:** hydroxytyrosol, cytoprotective effects, antioxidant, anti-inflammatory, anticancer, non-communicable diseases

## Abstract

Hydroxytyrosol (HT) ((3,4-Dihydroxyphenyl)ethanol) is a polyphenol mainly present in extra virgin olive oil (EVOO) but also in red wine. It has a potent antioxidant effect related to hydrogen donation, and the ability to improve radical stability. The phenolic content of olive oil varies between 100 and 600 mg/kg, due to multiple factors (place of cultivation, climate, variety of the olive and level of ripening at the time of harvest), with HT and its derivatives providing half of that content. When consumed, EVOO’s phenolic compounds are hydrolyzed in the stomach and intestine, increasing levels of free HT which is then absorbed in the small intestine, forming phase II metabolites. It has been demonstrated that HT consumption is safe even at high doses, and that is not genotoxic or mutagenic in vitro. The beneficial effects of HT have been studied in humans, as well as cellular and animal models, mostly in relation to consumption of EVOO. Many properties, besides its antioxidant capacity, have been attributed to this polyphenol. The aim of this review was to assess the main properties of HT for human health with emphasis on those related to the possible prevention and/or treatment of non-communicable diseases.

## 1. Introduction

**Figure 1:**
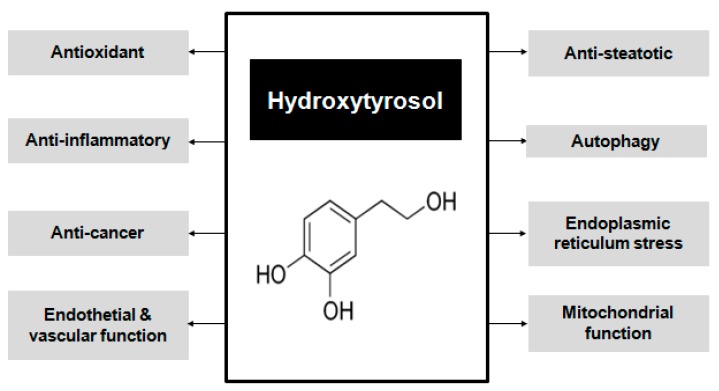
Principal cytoprotective beneficial effects of hydroxytyrosol.

According to the Global Health Observatory (GHO) of the World Health Organization (WHO), non-communicable diseases (NCDs), also known as chronic diseases, are the main cause of mortality worldwide. These illnesses include diabetes, hypertension, metabolic syndrome, non-alcoholic fatty liver disease, heart diseases, stroke, cancer, and chronic respiratory diseases, and it is estimated that 38 million of the 56 million global deaths in 2012 were due to NCDs [1,2]. NCDs are mostly characterized by mitochondrial alterations, impaired metabolism, oxidative stress, and inflammation [2], molecular and cellular alterations that are key issues to be dealt with for prevention and treatment [3]. In this context, hydroxytyrosol (HT) (3,4-Dihydroxyphenyl)ethanol; Figure 1) is a polyphenol mainly present in extra virgin olive oil (EVOO) [4] that has a potent antioxidant activity [5,6,7]. Moreover, HT provides other beneficial benefits that may be of importance for human health [8], namely anti-inflammatory [9] and anticancer [10] effects, enhancement of endothelial and vascular function [11], anti-steatotic [7,12] properties, and improvement of endoplasmic reticulum stress [13], autophagy [14] and mitochondrial function [15]. Interestingly, HT is the only polyphenol recognized by the European Food Safety Authority as a protector of low density lipoproteins (LDL) from oxidative damage [16], considering the finding that HT reduces the expression of adhesion molecules in endothelial cells, preventing the oxidation of LDL [17]. In order to obtain this effect, 5 mg of HT and its derivatives should be consumed in the form of extra virgin olive oil (EVOO) per day, which can be easily achieved in a healthy diet [16,18]. Moreover, HT exhibits a preventive effect on the metabolic syndrome in obese subjects, by decreasing fat deposition in liver and muscle, enhancing antioxidant capacity with both direct and indirect effects, and improving mitochondrial function. Furthermore, HT has a significant effect in attenuating glucose and lipid metabolic disorders in mice [19]. In view of these considerations, the aim of this review was to assess the main properties of HT for human health with emphasis on those related to the possible prevention and/or treatment of NCDs.

## 2. Material and Methods

The review included several searches that considered the metabolic and beneficial effects of HT in in vivo and in vitro models, using the PubMed database from the National Library of Medicine-National Institutes of Health. A particular emphasis was placed in the participation of HT as an anti-oxidant, anti-inflammatory, anti-cancerogenic, and anti-steatotic molecule, affording endothelial and vascular function, endoplasmic reticulum stress, autophagy, and mitochondrial function improvements.

## 3. General Characteristics of Hydroxytyrosol

Olive oil can be separated into a larger fraction and a smaller portion. The larger fraction (98–99% of total oil weight) corresponds to the saponifiable portion that is mainly composed of triglycerides (TGs), oleic acid being their most important component [4]. The minor fraction includes two types of components, namely, the unsaponifiable fraction and the soluble part, the latter containing the phenolic compounds that exhibit antioxidant activity, such as acids and alcohols, with HT belonging to the last group [4]. The olive oil phenols can be divided in three categories, simple phenols (HT and tyrosol), secoiridoids (SEC; oleuropein), and lignans [20]. HT can be present as a simple phenol or esterified with elenoic acid, forming oleuropein [4]. Both HT and tyrosol are formed from the hydrolysis of the SEC aglycones of oleuropein and ligstroside [20]. Hydrolysis of oleuropein takes place during the storage of olive oil and results in the formation of HT, tyrosol and ethanol. HT and oleuropein are catecholes [20], and it has been shown that the antioxidant effect of EVOO phenolic compounds is due to the presence of the catechol group [21]. The antioxidant properties of HT are related to hydrogen-atom donation and the ability to improve radical stability by forming an intramolecular hydrogen bond between the free hydrogens of their hydroxyl groups and their phenoxyl radicals [22]. The beneficial effects of HT have been demonstrated in human, cellular and animal models, mainly consumed as EVOO [17,23] The HT from EVOO has also shown to increase insulin sensitivity and the secretory capacity of β-pancreatic cells, thus reducing the risk of developing metabolic syndrome. The phenolic content of olive oil depends on the place of cultivation, climate, variety of the olive, and its level of ripening at the time of harvest, reaching levels of 100–600 mg/kg, and approximately 50% of that phenolic content corresponds to HT and its derivatives [24,25]. HT is also present in wine, and it is considered a secondary metabolite of tyrosine formed by yeasts during alcoholic fermentation, having an approximate concentration up to 5 mg/L [26]. In humans, there is an endogenous formation of HT from dopamine, which is favored in the presence of ethanol [24]. De la Torre et al., 2006 compared short-term and postprandial effects of moderate doses of EVOO (1.7 mg HT) and wine (0.35 mg HT), and found that the urinary recovery of HT was greater from wine intake than from EVOO consumption, despite having a lower HT content, which is explained by the endogenous formation from dopamine favored by the alcohol content of the wine [27]. EVOO’s phenolic compounds are rapidly hydrolyzed against alkaline conditions in the stomach and intestine, resulting in the increase of free HT to be absorbed in the small intestine where it forms phase II metabolites [11]. The in vitro colon fermentation of HT showed production of phenolic acids (phenylacetic and phenylpropionic acids) and degradation of HT-acetate and oleuropein by fecal culture medium, thus stabilizing HT and tirosol levels [28]. Furthermore, a moderate intake of a phenol-rich olive oil in males significantly raised the concentration in human feces of free HT [28]. These data suggest a role for colon microbiota in HT biotransformation, leading to an enhanced biodisponibility of the polyphenol [28], and that the cardioprotective effects of EVOO observed in hypercholesterolemic subjects could be contributed by the increased populations of bifidobacteria and in phenolic microbial metabolites with antioxidant properties [29]. HT and its metabolites accumulate in a dose-dependent manner in the liver, kidney and brain [30]. Auñon-Calles et al. [31] performed a toxicological evaluation of HT in rats, assessing doses of 5, 50 and 500 mg/kg/d for 13 weeks, which did not induce toxicologically relevant changes at any dose, proposing a no observed adverse effect level (NOAEL) of 500 mg/kg/d. Thus, HT consumption is safe even at high doses, without having genotoxic or mutagenic actions in vitro [32]. HT is mostly absorbed at the intestinal level, and is eliminated through urine, reaching a peak at 0–4 h after ingestion [33]. Miró-Casas et al. [34] investigated the absorption of tyrosol and HT after consumption of either a moderate dose (50 mL once) or sustained doses (25 mL/d for a week) of EVOO in humans. After both dosages, the urinary HT levels increased similarly with an estimated half-life of approximately 8 h, suggesting that urinary levels of HT are not a reliable biomarker of EVOO intake [34].

## 4. Protective Properties of Hydroxytyrosol

### 4.1. Antioxidant Effect

The antioxidant properties of HT are associated with its high degree of absorption and bioavailability, which is essential for its pharmacokinetic properties [35], and with its catecholic structure that allows (1) free radical scavenger and radical chain breakdown actions; and (2) a metal chelation feature reducing free-radical generation with production of very stable derivatives [36]. The assessment of the effects of a long-term diet (8 weeks) supplemented with HT (0.03 gm %) in C57BL/6 mice showed lower levels of plasma cholesterol and circulating leptin, in addition to a decreased glutathione disulfide (GSSG)/reduced glutathione (GSH) ratio in adipocytes [37]. Moreover, the HT-supplemented diet modulated gene expression of pathways related to oxidative stress (OS) in adipose tissue, particularly those of GSH and related enzymes [37]. The study of the effect of HT in rats with breast cancer and doxorubicin-induced cardiotoxicity revealed improvement in the drug-induced cardiac alterations by reducing mitochondrial damage and OS, suggesting that HT could enhance the electron transport chain [38]. Under OS conditions, the transcription factor nuclear erythroid 2-related factor 2 (Nrf2) is activated, a transcriptional regulator that controls the expression of phase II enzymes and of genes involved in oxidative defense. Mechanistically, Nrf2 is inactive in the cytoplasm due to the formation of a complex with its inhibitor Keap-1 [39]. Following the release of Keap-1 from complex induced by OS or electrophilic agents, Nrf2 is translocated to the nucleus, where it binds to promoters containing antioxidant response elements (AREs), resulting in the transactivation of the respective genes for phase II and antioxidant enzymes [39]. The effect of HT on pigment epithelial cells of the retina submitted to OS induced by acrolein indicated that HT was able to increase the translocation of Nrf2 to the nucleus, which resulted in increased protein expression and activity of γ-glutamyl cysteine ligase (GCL), NAD(P)H quinone oxidoreductase (NQO1), heme oxygenase-1 (HO-1), GSH reductase (GR), GSH peroxidase (GPx) and catalase (CAT) [40]. These results demonstrate that HT confers additional indirect antioxidant protection in addition to its already known direct antioxidant properties [40]. In addition, porcine pulmonary artery endothelial cells exposed to HT in the presence of H_2_O_2_ exhibited prevention of the intracellular increase of reactive oxygen species (ROS) levels through increased CAT mRNA and enhanced expression and activity of the transcription factor forkhead transcription factor 3a (FOXO3a), both at cytosolic and nuclear levels [41]. Activation of FOXO3a directly increased the expression of antioxidant enzymes, through the phosphorylation of AMP-activated protein kinase (AMPK) that translocates FOXO3a to the nucleus. Therefore, these findings demonstrate that HT positively regulates the antioxidant defense system in the cells studied [41] (Table 1).

### 4.2. Anti-Inflammatory Effect

HT has been described as one of the polyphenols of EVOO with the most potent anti-inflammatory effects, which involve (1) inhibition of nitric oxide (NO) and prostaglandin E2 (PGE2) production; (2) decreased secretion of pro-inflammatory cytokines (interleukin (IL)-1α, IL-1β, IL-6, IL-12, tumor necrosis factor-α (TNF-α) and chemokines such as C-X-C motif chemokine 10 (CXCL10)/interferon γ-induced protein 10 (IP-10), (C-C motif) ligand 2 (CCL2)/monocyte chemoattractant protein 1 (MPC-1); and (3) decreased gene expression of inducible NO synthase (iNOS), IL-1α, CXCL10/IP-10, macrophage inflammatory protein-1β (MIP-1β), matrix metalloproteinase-9, and prostaglandin E2 synthase (PGES) [9]. In this context, the study of the effect of HT supplementation (10 mg/kg/d) in rats with non-alcoholic fatty liver disease (NAFLD) induced by a high-fat diet (HFD) for 5 weeks showed increased levels of peroxisome proliferator-activated receptor-α (PPAR-α) and reduced expression of TNF-α and IL-6, thus confirming its anti-inflammatory properties [42]. Some pro-inflammatory cytokines, such as TNF-α and IL-1β and reactive oxygen species, have been shown to activate the redox-sensitive transcription factor nuclear factor-κB (NF-κB), which is crucial in a number of cellular processes such as inflammation, immunity, cell proliferation and apoptosis [43]. NF-κB activation is related to pro-inflammatory pathways, by triggering (1) the expression of genes encoding pro-inflammatory cytokines such as TNF-α, IL-1, IL-6 and IL-17, chemokines, and adhesion molecules [44]; and (2) the activation of inflammasome NLRP3 (nucleotide-binding oligomerization domain (NOD), leucine-rich repeat containing family, pyring domain containing 3) acting in the priming phase [45]. In agreement with this contention, HT supplementation in TNF-α-activated human umbilical vein endothelial cells (hECs) down-regulated NF-κB signaling by reducing protein levels of phosphorylated inhibitor of κB kinase αβ (IKKαβ), inhibitor of κBα (IκBα), and p65, which are crucial in the NF-κB pathway, supporting the role of NF-κB inactivation in the anti-inflammatory action of HT [46] (Table 1).

### 4.3. Anti-Cancerogenic Effect

The antioxidant, antiproliferative, pro-apoptotic, and anti-inflammatory properties attributed to HT suggest an anti-carcinogenic potential of the polyphenol [8], which are linked to the prevention of some types of cancer through different mechanisms. In the study of Warleta et al. [47], HT contribute to the preventive activity against breast cancer attributed to EVOO, through the reduction of OS and the protection of DNA oxidation in normal cells of the breast at physiological concentrations. Furthermore, the antitumor effect of HT could be exerted through down-regulation of epidermal growth factor receptor (EGFR) expression, as shown in human colon tumor cells in which HT induced ubiquitination and degradation of the receptor, effects that are associated with a decrease in cell proliferation, thus complying with its anti-cancerous effect [48]. Besides, Notarnicola et al. [49] studied the effect of HT on human colorectal cancer cells and found that HT had antiproliferative and pro-apoptotic effects. The inhibitory effects of cell growth are mediated through inhibition of fatty acid synthase (FAS), which has been found to be directly related to the aggressiveness of the tumor [49]. Moreover, HT has shown to inhibit the cell proliferation and induce apoptotic cell death in breast cancer cells, having a cytotoxic effect on MCF-7 cells in a dose dependent manner [50] (Table 1).

### 4.4. Effects on Endothelial and Vascular Function

In addition to the properties already discussed, HT has a beneficial effect on endothelial and vascular function [51,52]. Evaluation of endothelial function in a double-blind, randomized controlled clinical trial was performed in 13 patients with pre-hypertension and grade 1 hypertension who were given either functional olive oil (961 mg/kg phenolic compounds) or regular olive oil (289 mg/kg phenolic compounds) [51]. Data shown revealed that after 5 h of the intake, the functional olive oil showed significant differences with respect to the regular olive oil, namely, increased reactive hyperemia and plasma *C*_max_ of HT sulfate (metabolite), and decreased oxidized LDL, TG and glucose levels, with greater benefits for endothelial function than olive oil with a lower phenolic content [51]. Furthermore, a randomized controlled double-blind crossover trial in 18 apparently healthy volunteers who consumed one capsule of olive leaf extract (51 mg oleuropein, 10 mg HT) or control capsule in a single ingestion, revealed that extract consumption induced significant diminution in arterial stiffness, digital pulse volume, and IL-8 levels compared to control, with increased excretion of metabolites derived from oleuropein and HT [52]. Hence, phenolic compounds of olive leaf extract positively modulated vascular function and inflammation [50]. Additionally, HT and its metabolites significantly decreased secretion of E-selectin, P-selectin, intercellular adhesion molecule 1 (ICAM-1) and vascular cell adhesion molecule 1 (VCAM-1) in human aortic endothelial cells (HAEC) at physiological concentrations (1, 2, 5 and 10 μM) co-incubated with TNF-α for 18 and 24 h [11], concomitantly with reduction in markers of endothelial dysfunction that may contribute to the prevention of atherosclerosis [11]. Finally, the effect of HT in both free and complex forms (SEC) on the aortic and cardiac proteome of female Wistar rats, revealed a higher free HT content after supplementation with both forms of HT, together with the detection of the phase-II metabolite HT-3-*O*-sulfate, which appeared only after the diet with HT [53]. The proteome of both heart and aorta changed after supplementation with EVOO’s phenolic compounds, the difference being greater when supplementation was with SEC, which may be due to the higher HT content in the tissue after the SEC diet, with a negative regulation of proteins involved in blood vessel occlusion, endothelial cell proliferation, pro-inflammatory processes, and cell death [53] (Table 1).

### 4.5. Anti-Steatotic Effect

Supplementation with oleuropein, the HT precursor compound, has a protective effect in reversing the negative effects induced by a HFD in mice, regularizing hepatic steatosis, hyperlipidemia, and increased body weight and liver weight [54]. Also, the study of the effect of the different phenolic compounds (HT, tyrosol, and oleuropein) present in EVOO in rat hepatocytes revealed a decrease in de novo synthesis of fatty acids (FAs), cholesterol, and TG [55]. Moreover, in the study by Pirozzi et al. [42] using HT supplementation (10 mg/kg/d) in rats with HFD-induced NAFLD, protective effects against liver damage caused by HFD were observed, including an anti-inflammatory action, improved glucose tolerance, increased insulin sensitivity, decreased OS, and reduced plasma cholesterol, increasing the hepatic levels of PPAR-α (Table 1).

### 4.6. Effects on Endoplasmic Reticulum (ER) Stress and Autophagy

OS and high levels of saturated FAs induce protein misfolding or unfolding constituting an ER stress condition triggering the unfolded protein response (UPR) to recover protein homeostasis and stability [56]. However, if the latter feature is not reached, pathological responses can be attained, including several liver diseases [56]. It has been demonstrated that HT has an effect on regulating cell ER stress and autophagy. In fact, Giordano et al. [13] studied human hepatocarcinoma HepG2 cells with ER stress induced by tunicamycin and treated with HT at concentrations of 1 and 5 μM. At both HT concentrations, two markers of ER stress involved in the UPR, namely, ER CCAAT-enhancer-binding protein homologous protein (CHOP) and binding immunoglobulin protein (BiP), were significantly reduced over control values, as well as BiP’s transcription factor activating transcription factor 6α (ATF6α) that was reduced with the higher HT dose [13]. Furthermore, HT at both doses restored levels of B-cell lymphoma 2 (Bcl2), an anti-apoptotic protein, improving ER homeostasis [13]. Besides, the incubation of HepG2 cells with HT for 24 h (1 and 5 μM) decreased mRNA levels of ER CHOP and BiP, thus evidencing the preventive activity of HT against the ER stress in the hepatocyte [57].

Autophagy has been identified as a protective process during the development of osteoarthritis, which is critical for the survival of chondrocytes. Recently, Cetrullo et al. [14] studied the possibility of modulating autophagy in chondrocytes through the treatment with HT, by assessment of DNA damage and cell death in human C-28/12 chondrocytes undergoing primary osteoarthritis when exposed to hydrogen peroxide. HT increased autophagy markers and protected chondrocytes from DNA damage and cell death induced by OS [14]. Furthermore, in rats submitted to severe exercise for an 8-week period, supplementation with 25 mg/kg/day of HT reduced OS and thereby mitochondrial impairment, with reduction of muscular atrophy induced by autophagy and mitochondrial fission [58] (Table 1).

### 4.7. Effects on Mitochondrial Function

HT is known to improve mitochondrial function either by increasing the activity and/or expression of mitochondrial complexes I, II, III, IV, and V [15,38,59,60]. The 3T3-L1 adipocytes subjected to HT in the concentration range of 0.1–10 μM stimulated the expression and activity of peroxisome-proliferator activator receptor γ co-activator 1α (PGC1α), which promotes mitochondrial function by increasing the expression and activity of all complexes involved in the respiratory chain and oxidative phosphorylation [60]. Accordingly, HT was shown to improve the mitochondrial electron transport chain in rats with cardiotoxicity induced by doxorubicin by increasing the levels of complex-II and complex-III that partially restore the respiratory chain activity [38]. This effect was also observed in diabetic mice after a 2-month treatment with HT at doses of 10 and 50 mg/kg, where the expression levels of complexes I, II, and IV and the activity of complex I were increased in the brain, improving mitochondrial function [15]. In agreement with these views, HT also stimulates mitochondrial biogenesis in ARPE-19 human retinal pigment epithelial cells subjected to OS, through enhancement in the expression of PGC1α that leads to an increased protein expression of mitochondrial transcription factor A, uncoupling protein 2 (UCP2) and mitochondrial complexes [40] (Table 1).

### 4.8. Other Effects

In addition to the aforementioned effects, HT may have an effect at the bone level, decreasing bone loss in femur and formation of multinucleated osteoclasts. These actions were ascribed to the antioxidant effect of HT, taking into account that high levels of ROS suppress bone metabolism and increase the formation of osteoclasts, thus preventing bone mass loss [61].

Another interesting effect of HT is related to the management of pain related to inflammation, as shown by Takeda et al. [62] in a double-blind controlled study in which 25 adults with early osteoarthritis of the knee were given one tablet of 50 mg/day of olive extract (10 mg HT) or placebo for 4 weeks. A significant diminution in pain was observed in the group after 2 and 4 weeks of HT treatment, pointing to HT as effective treatment to reduce pain in patients with gonarthrosis [62].

Reestablishment of cognitive alterations provoked by prenatal stress is a further beneficial effect of HT (10 or 50 mg/kg/d), as shown in rats with alterations of learning capacity and memory performance by improving the alterations of the neural proteins such as brain-derived neurotrophic factor (BDNF), growth associated protein 43 (GAP43), synaptophysin, NMDAR1, NMDANR2A, NMDANR2B, while decreasing OS [63].

## 5. Conclusions

Data discussed on this review show that HT is a polyphenol which has not only powerful antioxidant activity but also possesses other cytoprotective properties with beneficial health effects. These include anti-inflammatory, anticancer, and anti-steatotic effects, with improvement of endothelial and vascular function, endoplasmic reticulum stress, autophagy, and mitochondrial function. Therefore, HT has a positive outcome on molecular and cellular alterations commonly associated with NCDs, becoming a potential therapeutic agent for the prevention and/or treatment of these chronic diseases. Interestingly, HT has been studied in cellular, animal and human models that demonstrate the safety of the polyphenol; nevertheless, more studies in humans are needed to determine the therapeutic doses. Main beneficial properties of HT and the metabolic pathways involved in these effects are summarized in Figure 1.

## Figures and Tables

**Table 1:** Summary of hydroxytyrosol (HT) properties in different study models. GCL: γ-glutamyl cysteine ligase; GR: GSH reductase; HO-1: heme oxygenase-1; NQO1: NAD(P)H quinone oxidoreductase; CAT: catalase; ROS: reactive oxygen species; FOXO3a: forkhead transcription factor 3a; NO: nitric oxide; PGE2: prostaglandin E2; iNOS: inducible NO synthase; PGES: prostaglandin E2 synthase; NAFLD: non-alcoholic fatty liver disease; HFD: high-fat diet; PPAR-α: peroxisome proliferator-activated receptor-α; GSH: glutathione-ethyl ester; CHOP: CCAAT-enhancer-binding protein homologous protein; BiP: binding immunoglobulin protein; hECs: human umbilical vein endothelial cells; IL: interleukin; EGFR: epidermal growth factor receptor; LDL: low density lipoproteins; UPR: unfolded protein response.

Properties	Reference	Model	Intervention	Results/Findings
Antioxidant	Giordano et al., 2014 [37]	Animal/cellular	C57BL/6 mice were randomly assigned to a standard diet without HT or the same diet supplemented with HT (0.03 gm%) for 8 weeks. Murine 3T3-L1 pre-adipocytes, after differentiation, were cultured with H_2_O_2_ and 1 or 5 μM HT.	Modulated gene expression of pathways related to oxidative stress (OS) in adipose tissue. Decreased GSSG/GSH ratio in cultured adipocytes.
	Granados-Principal et al., 2014 [38]	Animal	Thirty-six female Sprague–Dawley rats with induced mammary tumors were divided into four groups: control, HT (0.5 mg/kg, 5 days/week), doxorubicin (1 mg/kg/week), and doxorubicin + HT.	Improved drug-induced cardiac alterations induced by doxorubicin by reducing mitochondrial damage and OS.
	Zhu et al., 2010 [40]	Cellular	Cultured ARPE-19 cells were pretreated with HT dissolved in dimethylsulfoxide (DMSO; final DMSO concentration ≤ 0.025%) and treated with acrolein to induce OS.	Increased translocation of Nrf2 to the nucleus in cells not challenged with acrolein. Increased expression of phase II detoxifying enzymes (GCL, GR, GSH GPx, HO-1, NQO1) and of genes involved in oxidative defense (indirect effect). Increased expression and activity of CAT.
	Zrelli et al., 2011 [41]	Cellular	VECs with OS induced by H_2_O_2_ were incubated with HT (10, 30 and 50 μM).	Prevented intracellular increase of ROS levels. Increased CAT mRNA expression levels. Increased expression and activity of FOXO3a.
Anti-inflammatory	Richard et al., 2011 [9]	Cellular	Murine macrophages (RAW264.7 cells) were stimulated with lipopolysaccharide (LPS) and treated with olive vegetation water containing 2.5% HT.	Inhibited of NO and PGE2 production. Decreased secretion of cytokines (interleukin (IL)-1α, IL-1β, IL-6, IL-12, TNF-α, and chemokines (CXCL10/IP-10, CCL2/MPC-1)). Decreased gene expression of iNOS, IL-1α, CXCL10/IP-10, MIP-1β, matrix metalloproteinase-9 and PGES.
	Pirozzi et al., 2016 [42]	Animal	Rats with NAFLD were divided into three groups: Control diet, HFD and HFD + HT (10 mg/kg/d) for 5 weeks.	Increased levels of PPAR-α. Reduced the expression of pro-inflammatory cytokines TNF-α and IL-6.
	Lopez et al., 2017 [46]	Cellular	hECs were cultured in the presence of HT and HT metabolites and treated with TNF-α.	HT and its metabolites suppressed production of ROS induced by TNF-α. Down-regulated TNF-α-induced phosphorylation of NF-κB signaling proteins (IKKαβ, IκBα, and p65) in hECs.
Anticancer	Warleta et al., 2011 [47]	Cellular	Three human breast cell lines were treated with HT and tyrosol: mammary epithelial cells (MCF10A) and breast cancer cells (MDA-MB-231, MCF7).	Decreased ROS production in a dose-dependent manner in MCF10A cells. Decreased H_2_O_2_ induced ROS level in breast cancer cells. Reduced DNA damage significantly in MCF7, MDA-MB-231 and MCF10A (in unexposed cells).
	Terzuoli et al., 2016 [48]	Cellular	Human colorectal adenocarcinoma cells (HT-29, CaCo2, WiDr) or human colon fibroblast cells (CCD18Co) treated with HT.	Down-regulated EGFR expression in colon tumor cells by promoting its degradation via lysosomal and proteasomal mechanisms.
	Notarnicola et al., 2011 [49]	Cellular	Human colon adenocarcinoma cell lines (HT-29 and SW-620) were treated with HT and oleuropein at different concentrations (10, 25, 50 and 100 μM) for 24 and 72 h.	Reduced both gene expression and activity of FAS (starting from 10 μM). Showed an anti-proliferative effect in SW-620 both cell lines Had a pro-apoptotic effect in HT-29 cells.
	Han et al., 2009 [50]	Cellular	MCF-7 human breast cancer cells were treated with HT (6.25, 12.5, 25, 50 μg/mL) and oleuropein for 12 h.	Inhibited cell proliferation, reducing the cell viability in a time and concentration-dependent manner. Induced cell apoptosis by caspases activation and also blocked the cell cycle in G1 phase.
Endothelial and vascular function	Valls et al., 2015 [51]	Human	Thirteen pre- and stage-1 hypertensive patients received a single dose of 30 ml of functional virgin olive oil (FVOO) (phenolic content = 961 mg/kg) or regular virgin olive oil (VOO) (phenolic content = 289 mg/kg) in a postprandial randomized, double blind, crossover trial.	FVOO improved human endothelial function (ischemic reactive hyperemia values were higher with FVOO than with VOO). Postprandial values of PAI-I, and hsCRP were lower after FVOO versus VOO. FVOO reduced oxidized LDL.
	Lockyer et al., 2015 [52]	Human	Eighteen healthy volunteers who consumed either OLE (51 mg oleuropein; 10 mg HT), or a matched control (separated by a 4-week wash out) on a single occasion were studied in a randomized, double-blind, placebo-controlled, cross-over, acute intervention trial.	OLE reduced arterial stiffness. OLE reduced IL-8 production.
	Catalán et al., 2015 [11]	Cellular	Human aortic endothelial cells (HAEC) were treated with HT and the mixture of its metabolites (1, 2, 5, and 10 μM) and co-incubated with TNF-α for 18 and 24 h.	HT and its metabolites reduced the secretion of E-selectin, ICAM-1, and VCAM-1. Free HT and HT metabolites were effective in the reduction of the endothelial dysfunction biomarkers.
	Catalán et al., 2016 [53]	Animal	Twelve female Wistar rats were separated in three groups: standard diet, diet supplemented with HT or diet supplemented with secoroids (SEC) in doses of 5 mg/kg/d for 21 days.	HT was detected in heart tissue mainly in its free form after supplementation with HT or SEC. Supplementation changed heart and aorta proteome. These proteins are related to cardiovascular function, improving endothelial and vascular function.
Anti-steatotic	Park et al., 2011 [54]	Animal	Male C57BL and 6N mice were separated into three groups: normal diet, HFD, HFD supplemented with oleuropein (the precursor of HT), for 10 weeks.	Had a protective effect in reversing the negative effects induced by a HFD, regularizing: hepatic steatosis, increased plasma lipids, and increased body weight and liver. Down-regulated transcription factors and their target genes involved in adipogenesis.
	Priore et al., 2014 [55]	Cellular	Rat-liver cells were treated with HT, tyrosol and oleuropein (EVOO phenols).	Cholesterol synthesis and fatty acids (FA) synthesis were inhibited by the treatment. Reduced the activity of ACC.
	Pirozzi et al., 2016 [42]	Animal	Male rats were divided into three groups: standard diet, HFD, HFD + HT (10 mg/kg/d) for 5 weeks.	Reduced AST, ALT and cholesterol levels in serum, and reduced liver steatosis. Increased the activity of PPAR-α. Increased phosphorylation of ACC, increasing hepatic metabolism and oxidation of FA.
ER stress and autophagy	Giordano et al., 2014 [13]	Cellular	Human hepatocarcinoma cells (HepG2) were treated with HT (1 μM and 5 μM) and 100 μM lipoic acid (LA) and glutathione-ethyl ester (GSH), for 24 h. UPR was induced tunicamycin for 4 h.	Reduced mRNA levels of CHOP and BiP compared with LA and GSH and with tunicamycin alone. Reduced protein levels of BiP and levels of eIF2a. Modulated the antiapoptotic Bcl2 protein levels. Prevented ER stress in hepatic cells.
	Giordano et al., 2015 [57]	Cellular	Human hepatocarcinoma cells (HepG2) were treated with two HT hepatic metabolites: i.e., 3-*O*-HT glucuronide and 4-*O*-HT glucuronide. UPR was induced tunicamycin.	Both metabolites reduced mRNA expression levels of CHOP and BiP. The treatment also decreased BiP and CHOP protein levels.
	Cetrullo et al., 2016 [14]	Cellular	Primary cultures of chondrocytes obtained from patients with knee arthroplasty were incubated in the absence or presence of 100 μM H_2_O_2_ and treated with HT.	Enhanced SIRT-1 expression, positively regulating autophagy.
	Feng et al., 2011 [58]	Animal	Eight-week-old male Sprague–Dawley rats were selected for the experiment by their ability to perform 1 week of running exercise at low speed. Rats were divided into four groups: sedentary with or without HT, and endurance exercise with or without HT (25 mg/kg/d). After eight weeks of exercise the analyses were done.	Reduced OS and thereby mitochondrial impairment. Reduced muscular atrophy induced by autophagy and mitochondrial fission and decreased expression of PGC-1α. Up-regulated autophagy.
Mitochondrial function	Hao et al., 2010 [60]	Cellular	Murine 3T3-L1 pre-adipocytes were treated with HT 0.1–50 μmol/L.	Stimulated activation and expression of PGC1α. Increased the mRNA expression levels of *Nrf1*, *Nrf2* and *Tfam* (1 μmol/L HT). Promoted protein expression of complex I, II, III and V. Promoted the activity of complexes I, II, III, IV and V, increasing oxygen consumption in adipocytes. Increased mitochondrial mass.
	Granados-Principal et al., 2014 [38]	Animal	Thirty-six female Sprague–Dawley rats with induced mammary tumors were divided into four groups: control, HT (0.5 mg/kg, 5 d/week), doxorubicin (1 mg/kg/week), and doxorubicin + HT.	Improved the mitochondrial electron transport chain in rats with cardiotoxicity induced by doxorubicin. Increased complexes II and III protein concentrations.
	Zheng et al., 2015 [15]	Animal	Male db/db C57BL/6J mice were separated into three groups: control, HT (10 mg/mg/d) and HT (50 mg/kg(d)	Improved expression of complexes I, II, and IV. Increased activity of complex I. Induced phase II antioxidant systems and inhibited protein oxidation in mice brain. Increased the expression of p-AMPK/AMPK, PGC-1α and SIRT1.
	Zhu et al., 2010 [40]	Cellular	Human retinal pigment epithelial cells (ARPE-19) were incubated with acrolein. The protective effects of HT were studied by pre-treating cells with HT for 48 h, followed by 24-h acrolein treatment in the absence of HT.	Increased the expression of PGC1α. Increased protein expression of mitochondrial transcription factor A and uncoupling protein 2 (UCP2). Increased the expression of complexes. Increased Nrf2 nuclear protein levels and its nuclear translocation. Enhanced phase II detoxifying enzymes.
